# Gait can reveal sleep quality with machine learning models

**DOI:** 10.1371/journal.pone.0223012

**Published:** 2019-09-25

**Authors:** Xingyun Liu, Bingli Sun, Zhan Zhang, Yameng Wang, Haina Tang, Tingshao Zhu

**Affiliations:** 1 Institute of Psychology, Chinese Academy of Sciences, Beijing, China; 2 Department of Psychology, University of Chinese Academy of Sciences, Beijing, China; 3 Department of Social and Behavioural Sciences, City University of Hong Kong, Hong Kong, China; 4 School of Computer Science and Technology, University of Chinese Academy of Sciences, Beijing, China; 5 School of Artificial Intelligence, University of Chinese Academy of Science, Beijing, China; Northeastern University, UNITED STATES

## Abstract

Sleep quality is an important health indicator, and the current measurements of sleep rely on questionnaires, polysomnography, etc., which are intrusive, expensive or time consuming. Therefore, a more nonintrusive, inexpensive and convenient method needs to be developed. Use of the Kinect sensor to capture one’s gait pattern can reveal whether his/her sleep quality meets the requirements. Fifty-nine healthy students without disabilities were recruited as participants. The Pittsburgh Sleep Quality Index (PSQI) and Kinect sensors were used to acquire the sleep quality scores and gait data. After data preprocessing, gait features were extracted for training machine learning models that predicted sleep quality scores based on the data. The *t*-test indicated that the following joints had stronger weightings in the prediction: the Head, Spine Shoulder, Wrist Left, Hand Right, Thumb Left, Thumb Right, Hand Tip Left, Hip Left, and Foot Left. For sleep quality prediction, the best result was achieved by Gaussian processes, with a correlation of 0.78 (*p <* 0.001). For the subscales, the best result was 0.51 for daytime dysfunction (p < 0.001) by linear regression. Gait can reveal sleep quality quite well. This method is a good supplement to the existing methods in identifying sleep quality more ecologically and less intrusively.

## Introduction

People spend almost one-third of their lifetime sleeping [1]. Adequate sleep is an important prerequisite for good health, while bad sleep can result in bad moods, inattention, fatigue, cardiovascular disease and even mortality [2–5]. Currently, people pay considerable attention to their sleep quality. However, to improve one’s sleep quality, people first need to know the exact condition of his/her sleep; that is to say, they need methods to monitor their sleep conditions.

New technologies provide convenient methods for people to self-monitor and improve their sleep in their daily life [6, 7]. Sleep quality can be assessed by objective physical indicators and behavior or subjective perception. Polysomnography (PSG) has been widely used for evaluating sleep in clinical research, but it has several disadvantages, such as being expensive, intrusive, time consuming and impractical [8]. Smartphones and smart bracelets can also be used for sleep activity monitoring. However, these devices are obtrusive and cumbersome since the user has to wear them or put them close to his or her body while sleeping [9]. In addition, these technologies can only be used to measure the sleep quality of people that are sleeping, making them inconvenient to operate in some situations; for example, these technologies cannot be used in physical examinations in the general population.

In contrast to objective assessments, self-report questionnaires such as the Pittsburg Sleep Quality Index (PSQI) are always used to measure subjective sleep quality. Landry, Best [10] found that perceived sleep quality is quite different from an objective measurement. In addition to the objective state, one’s subjective opinion of his or her own status is also of great value [11]. However, self-reported sleep quality is not reliable in some scenarios, such as when subjective sleep quality needs to be assessed without intrusion or the sleep quality of athletes or patients need to be assessed every day. In such cases, self-reported sleep quality might not work very well. In this paper, we propose to measure sleep quality using behavioral indicators instead.

With developments in computer science, machine learning models have been applied to predict sleep [9, 12–14]. The authors of these studies all agree that machine learning models can predict sleep well, even better than traditional logistic regression [13]. However, to our knowledge, all existing research in this area has collected data with the assistance of mobile phones or wearable sensors [9, 12–14]. Although machine learning models are cutting-edge tools used to analyze data, mobile phones and wearable sensors are obtrusive and cumbersome, as previously mentioned. Therefore, in this paper, we propose to use Microsoft Kinect, which is a noninvasive tool, to collect data.

Gait reflects how one walks and moves, and it can manifest one’s psychological and health conditions [15–17]. Sleep and gait can influence each other. On the one hand, it has been confirmed by many researchers that abnormal sleep has an impact on gait [18–20]. For example, a large-scale study measured individuals’ sleep conditions by ACTi graphs, and the study revealed that the gait speed of those who slept less than 6 hours per night was 3.5% slower than those who slept 6–6.8 hours per night [18]. This phenomenon was further confirmed by other researchers in older people [19] and in Parkinson’s disease patients [20]. On the other hand, gait has been shown to have an impact on sleep [21]. A prospective open-label study demonstrated that walking 10000 steps a day for 4 weeks can significantly improve sleep quality [21]. Similarly, sleep quality has been shown to have an impact on daily energy expenditure [22], and daily energy expenditure has been shown to be highly correlated with gait [23]. Thus, we expect that sleep can be measured by gait in a more ecological, less intrusive way. Microsoft Kinect may bring light to this field. This device can capture the 3-dimensional accelerations of the 25 main body joints with a 30 *Hz* sampling rate [24]. Not only is the Kinect noninvasive, low-cost and easy-to-use, but it has also been affirmed to be compatible in capturing real-time gait patterns in daily and clinical environments in earlier studies [17, 25–27].

In this paper, we used the Microsoft Kinect to capture gait data and trained machine learning models to automatically evaluate sleep quality.

## Methods

### Subjects

We recruited 59 participants. The participants were all first-year postgraduate students from the University of the Chinese Academy of Sciences and participated in this study in exchange for 200 RMB reimbursement. The consent was informed, and all of the participants signed written informed consent forms before the formal experiment. Of these participants, no participants were excluded for an injury or disability that affected their walking ability, but 3 students were excluded because of absences at appointments. In total, 56 participants were included in this study (24 males and 32 females 20; mean age = 24.42 years, SD = 1.61 years).

### Materials and instruments

The Pittsburgh Sleep Quality Index (PSQI) has been used to measure subjects’ sleep quality worldwide [28]. The questionnaire consists of 19 items and seven subscales: subjective sleep quality (SQ), sleep latency (SL), sleep duration (SD), habitual sleep efficiency (HSE), sleep disturbance (SDI), use of sleep medication (SM), and daytime dysfunction (DD) [28]. The range of the global score is from 0 to 21, and each subscale has a possible score of 0–3; a higher score indicates worse sleep quality.

**Fig 1:**
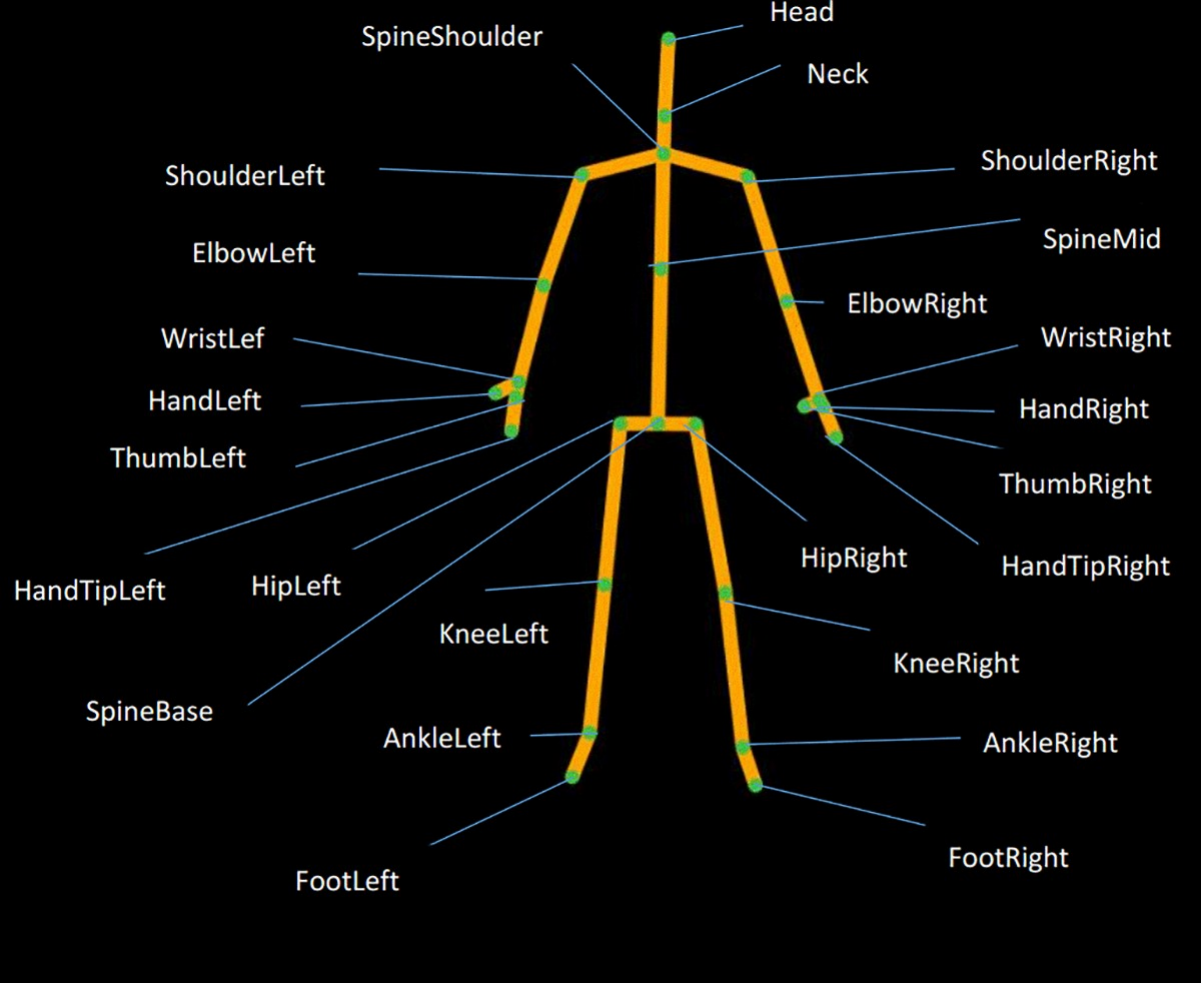
The 25 joints captured by a Kinect sensor.

We used two Kinect sensors to capture the gait data of the participants. Every second, the sensors were able to track the 3D body movement patterns of six individuals at once in 30 frames. Each frame contained the 3D coordinates of 25 joints of the body (Fig 1), including the Head, Neck and others.

### Procedure

In a noiseless room, participants first completed the PSQI and some other questionnaires so that they could determine the objective of the research. Then, the participants were instructed to move freely on a rectangular carpet (6 m × 1 m) for 2 minutes as they usually did during the free practice session. During the experiment, two Kinect sensors were placed on a diagonal line on the carpet to record the participants’ gait data. The study was approved by the Institutional Review Board of the Institute of Psychology at the Chinese Academy of Sciences (approval number: H15010).

## Results

### Data collection

The mean score of the PSQI was 7.32 (SD = 3.77). We collected 3600 frames of gait data that lasted 2 minutes for every participant (30 * 60 * 2 = 3600).

### Data preprocessing

We first ran a Gaussian filter (i.e., low-pass filter) [29] on the data for every dimension (X, Y, and Z) of the 25 joints to remove noisy data. The window length was 5, and the convolution kernel of the Gaussian filter was c = [1, 4, 6, 4, 1]/16. The formula was as follows:

$$Out[i]=\frac{1}{16}(In[i]\times 1+In[i+1]\times 4+In[i+2]\times 6+In[i+3]\times 4+In[i+4]\times 1)$$ (1)

**Fig 2:**
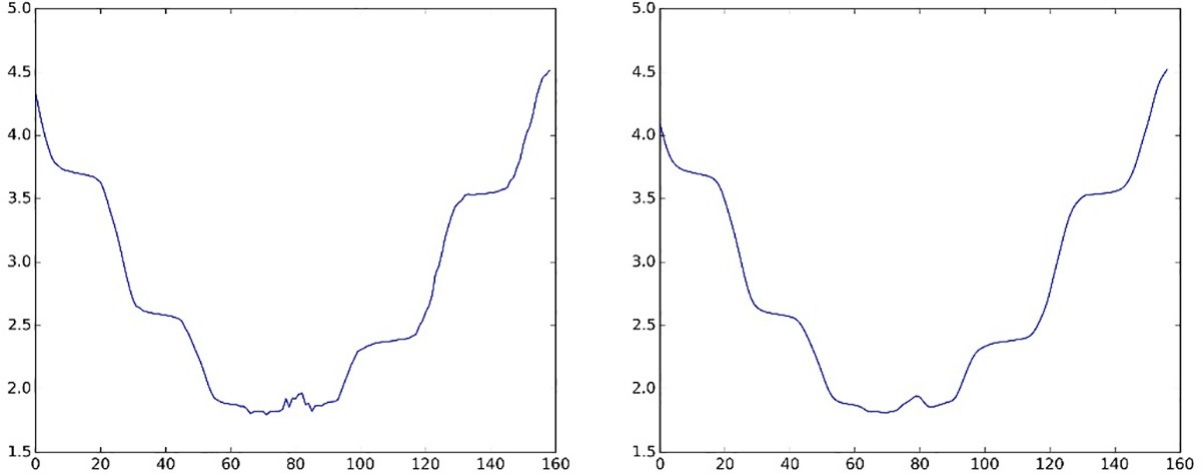
A comparison of the signal before and after the Gaussian filter.

The “In” corresponded to the original data captured by Kinects, and the “Out” corresponded to the new data. We took the Z-axis data of the SpineBase joint as an example. After noise reduction was performed by the Gaussian filter, the new Z-axis data (see Fig 2 right) were smoother than the original Z-axis data (see Fig 2 left).

Second, we used the Spine Base joint as the reference point in every frame to adjust the 3D coordinates of the different participants into the same coordinate system, which eliminated the differences in the relative positions of the participants and the Kinect. Specifically, the new 3D coordinates of each joint were calculated by subtracting the 3D coordinates of the Spine Base joint from the original coordinates of each joint. The other 24 joints were used to extract features.

Finally, we divided the participants gait data into segments because the Kinect could not capture the joint coordinates accurately when the participants were turning a corner. Therefore, we excluded the frames in which the participants were turning and divided one trial into several straight-walking segments. Therefore, there remained two kinds of segments relative to the Kinects’ location: face-towards and back-towards. We used the face-towards segments for feature extraction because the accuracy was better than that with the back-towards segments. Since gait is a cyclic physical activity, we only needed to extract a cycle of data for analysis. In addition, the fast Fourier transform for feature extraction requires a power of 2 data lengths; otherwise, the operation speed will be slow. Therefore, we choose 64 frames (approximately 2 s) as the length of the final data segment used in feature extraction. The selection process was as follows: we randomly selected 64 continuous frames of valid data from the 3600 frames as the final sample; that is to say, for each participant, we chose 64 frames, and the final number of segments was 3584 (64 * 56 = 3584).

### Feature extraction

We extracted the amplitudes of the fast Fourier transform (FFT) [30] from every face-towards segment, which is a method that has been widely used [31–33]. FFT transformed the sampled function from the time domain to the frequency domain for every dimension. For every joint in the gait data, we selected the 64 amplitude coefficients of every dimension as the features. The formula is:

$$X_{k}=\sum_{n=0}^{N-1}x_{n}e^{-i2\pi k\frac{n}{N}}\;k=0,\ldots ,N-1$$ (2)

The “N” corresponded to the segment length, and the “*x_n_*” corresponded to the gait data.

### Feature selection

We ran the Z-score function for feature normalization. Dimension reduction was performed before the model was trained because the high-dimensional feature vector was redundant. We first calculated the Pearson correlation coefficients between sleep quality and the extracted features. The Pearson correlation coefficient is a measure of the strength and direction of the linear relationship between two variables that is defined as the covariance of the variables divided by the product of their standard deviations [34]. We selected the 5 features with the highest absolute values of the correlation coefficients of every dimension to train the machine learning model. S1 Table shows the top 5 highest absolute values of the correlation coefficients of every dimension in sequence. The *p*-value is defined as the probability, under the null hypothesis H about the unknown distribution F of the random variable X, for the variate to be observed as a value equal to or more extreme than the value observed. A smaller *p*-value corresponds to a higher significance because it tells the investigator that the hypothesis under consideration may not adequately explain the observation [35]. Finally, we obtained 5 features for every dimension of every joint, i.e., 360 features in total (5*3*24 = 360).

**Fig 3:**
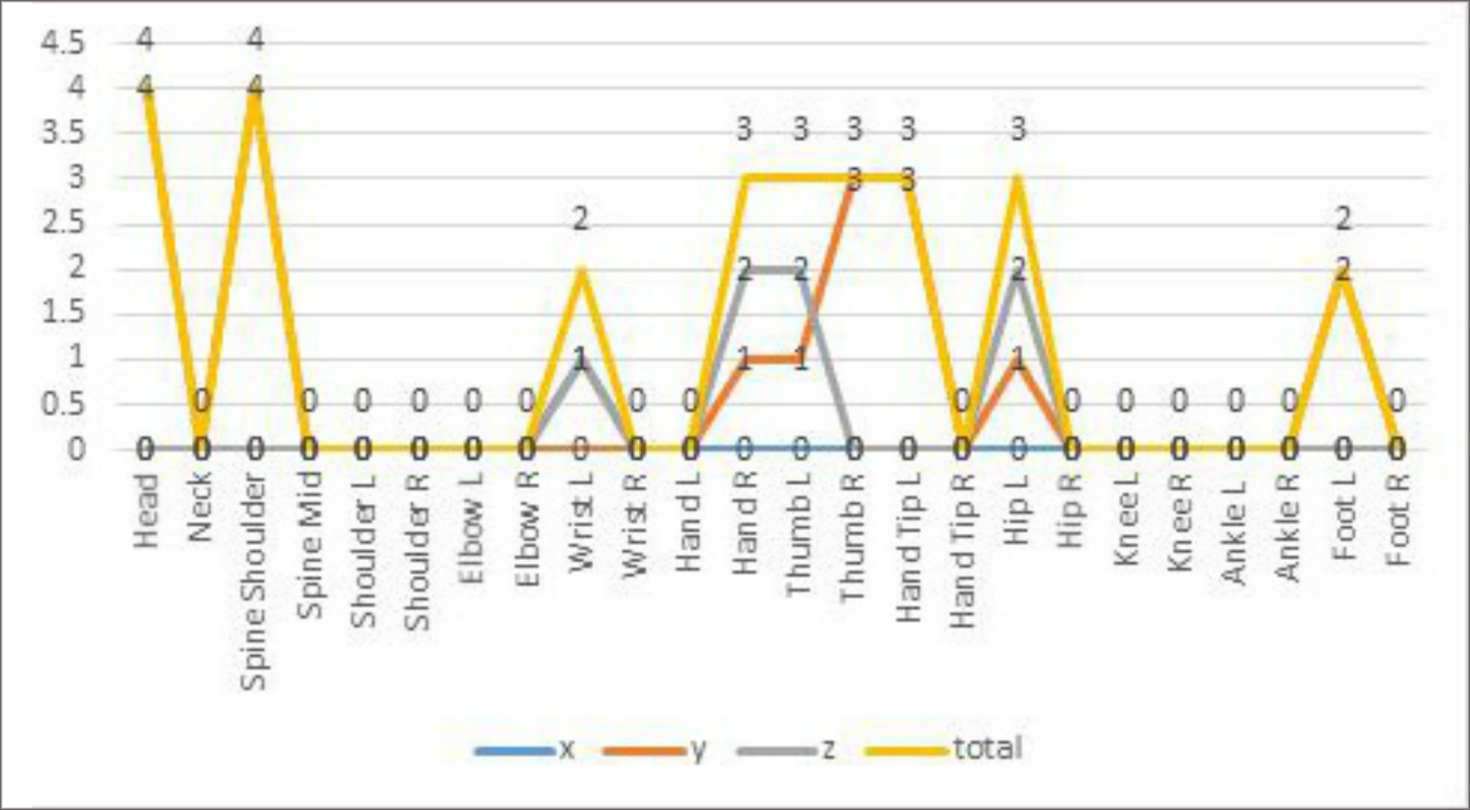
The number of features that were significantly different between the two groups.

To determine which features had a stronger weight in assessing sleep quality, we grouped participants into high-score (top 27%) and low-score (bottom 27%) groups by their PSQI scores. An independent samples *t*-test was used to compare the differences between the two groups. The *t*-test can be used to determine if the means of two sets of data are significantly different from each other. The t-score was defined as the mean difference in the variables all divided by the standard deviation divided by the square root of the sample size [36]. We selected 5 features from every dimension of every joint, i.e., 15 features for every joint. After the FFT was performed, it was difficult to tell the specific meaning of every feature, but it was clear that some points had strong weightings in assessing sleep quality. As shown in Fig 3, the following joints had 2 to 4 features significantly related to sleep quality: the Head, Spine Shoulder, Wrist Left, Hand Right, Thumb Left, Thumb Right, Hand Tip Left, Hip Left, Foot Left.

### Model training

We used WEKA, a set of machine learning algorithms that includes linear regression (LR), simple linear regression (SLR), Gaussian processes (GP), epsilon-support vector regression (E-SVR), and nu-support vector regression (N-SVR), and the 360 features to train the machine learning models with the default parameters in WEKA 3.7 or 3.8 (see S1 File) and 10-fold cross validation. To be specific, we randomly selected nine-tenths of the dataset as the training data and the remaining data as the testing data, and then we repeated the process ten times for every model. This method can avoid problems such as overfitting or selection bias to some degree [37].

The models were rated by the correlation coefficients between the predicted sleep quality score and the self-report score. The results are shown in Table 1.

**Table 1:** The correlations between the model-predicted scores and the self-reported sleep quality scores.

	LR ^a^	SLR ^b^	GP ^c^	E-SVR ^d^	N-SVR ^e^
**Total score**	0.77^***^	0.21	0.78^***^	0.2	0.46^***^
**Subjective sleep quality**	0.15	0.03	0.21	0.25	0.25
**Sleep latency**	0.15	0.03	0.15	-0.17	-0.22
**Sleep duration**	0.43^**^	0.18	0.27^*^	0.19	0.19
**Habitual sleep efficiency**	0.27^*^	0.42^**^	0.27^*^	0.39^**^	0.35^**^
**Sleep disturbance**	0.25	0.28^*^	0.29^*^	0.19	0.25
**Sleep medication**	——	——	——	——	——
**Daytime dysfunction**	0.51^***^	-0.09	0.44^***^	0.44^***^	0.43^***^

^a^ LR = linear regression

^b^ SLR = simple linear regression

^c^ GP = Gaussian processes

^d^ E-SVR = epsilon-support vector regression

^e^ N-SVR = nu-support vector regression

*** p < 0.001

** p < 0.01

* p < 0.05

For the total score, the best performance was achieved by GP with a score of 0.78 (p < 0.001). For the subscales, the best performance occurred with LR predicting DD (r = 0.51, p < 0.001), followed by LR predicting SD (r = 0.43, p < 0.01), SLR predicting HSE (r = 0.42, p < 0.01), and GP predicting SDI (r = 0.29, p < 0.05). All participants received a zero on the use of sleep medication (SM) subscale, so the prediction was invalid for this component.

## Discussion

We used Kinect to collect gait data on 56 participants and extracted features to build a machine learning model to reveal an individual’s sleep quality. The correlations were as high as 0.78, which was achieved by the GP model. The correlation coefficient value of 0.78 indicates a high positive correlation, which is relatively rare according to previous studies [38]. This result is consistent with an earlier study showing that sleep is associated with gait [18] and demonstrates that gait patterns can reveal sleep quality quite well. More importantly, a real-time prediction of human health status (sleep quality) scores can be implemented by using Kinect. As we can see from our experiment, participants only need to walk for two minutes, and then we can predict their sleep quality quite precisely. Therefore, Kinect is indeed a more convenient and less intrusive tool for measuring an individual’s sleep quality.

Since every subscale measured a different aspect of sleep quality [11], we explored the performance of the gait model to predict component scores. The DD, which reflects individuals’ difficulties in daytime activities, obtained the best predictive result by the gait model. Three other components, the SD, HSE and DSI, which are factors directly related to daytime performance, obtained significant predictive results by the gait models. However, the SQ and the SL cannot be predicted by gait data. One possible reason for this result is that SQ and SL affect people’s gait less than the other factors; after all, gait is an objective behavioral factor, and SQ and SL are factors that rely more on subjective feelings than do DD, SD, HSE and DSI, which have objective indicators.

Because of the nature of the FFT, it is difficult to explain the meaning of every feature; joints such as Head, Spine Shoulder, Wrist Left, Hand Right, Thumb Left, Thumb Right, Hand Tip Left, Hip Left, Foot Left have 2 to 4 features that revealed sleep quality significantly. This finding leads us to the conclusion that the shoulder and the above joints have stronger weightings.

The new method can measure sleep when people are awake in real-time and remotely, and these advantages allow us to measure people’s sleep conditions and obtain scores every day in a very short period of time without disturbances, which cannot be achieved by a questionnaire. Although the scoring process of the PSQI can be finished in several minutes, the PSQI still requires the recording of responses as well as calculations, which can possibly be viewed as burdensome [11]. For example, when we need to continually monitor the sleep quality of individuals, such as athletes, astronauts, and patients in a hospital, it is not suitable to ask them fill out questionnaires every day, and it is expensive to ask everyone wear a smart bracelet. Instead, we can obtain their sleep quality after asking them walk for a few minutes. Furthermore, because the Kinect is cheap and easy to obtain, this technology can also be applied in daily life. For example, this technology can be used in hospitals or in special conditions where a simple and quick method is needed for a large-scale survey, such as for physical examinations for everyone every year. Assessing people by their gait also has the advantage to be used remotely, while other biometrics might become obscured [39]. This method enhances the ecological validity in measuring sleep quality. Moreover, for those who are unable to finish the questionnaires, such as children, older individuals or illiterate individuals, an assessment by gait might be an alternative method.

Moreover, former studies mostly related gait speed [18, 40, 41] to sleep quality, which is an important factor, but there are more factors that can be used. In our study, we extracted 360 features to analyze people’s gait patterns, which is an advantage of using Kinect to observe people’s gait patterns.

One limitation is that we only used the PSQI to test the models. More criteria can be included in future studies. Another limitation is that there may exist a joint effect in the process of feature extraction. Another more sophisticated feature selection approach will be applied in our future study. Moreover, participants are all graduate students, and the sample population is not sufficiently large. In future studies, we plan to include large sample populations with individuals of different occupations, ages and cultural groups.

## Conclusion

This study provides an innovative method to measure sleep quality. The procedure of gait data collection is nonintrusive and ecological, and the results indicate that gait patterns can reveal sleep quality quite well. This method can be a supplementary method to the existing sleep quality measurements.

## Supporting information

S1 Table The highest absolute values of the correlation coefficients between sleep quality and the extracted features of every dimension. (DOCX)

S1 File The default parameters in WEKA 3.7 or 3.8. (DOCX)

S2 File Processed data. (CSV)
