# Supplementary material for: Gait can reveal sleep quality with machine learning models
Source: PLoS One. 2019 Sep 25;14(9):e0223012. doi: 10.1371/journal.pone.0223012 (PMC6760789; doi:10.1371/journal.pone.0223012)
Supplement: S1 Table — (DOCX) [file pone.0223012.s001.docx]

| **Joints** | **X/Y/Z-axis** | **Correlation coefficient** |
| --- | --- | --- |
| **AnkleRight** | X_1 | -0.26 |
| **AnkleRight** | X_2 | -0.26 |
| **AnkleRight** | X_3 | -0.25 |
| **AnkleRight** | X_4 | -0.25 |
| **AnkleRight** | X_5 | -0.22 |
| **AnkleRight** | Y_1 | 0.30*^a^ |
| **AnkleRight** | Y_2 | -0.24 |
| **AnkleRight** | Y_3 | -0.24 |
| **AnkleRight** | Y_4 | -0.23 |
| **AnkleRight** | Y_5 | -0.23 |
| **AnkleRight** | Z_1 | 0.43***^b^ |
| **AnkleRight** | Z_2 | 0.43*** |
| **AnkleRight** | Z_3 | 0.14 |
| **AnkleRight** | Z_4 | 0.14 |
| **AnkleRight** | Z_5 | 0.08 |
| **AnkleLeft** | X_1 | -0.31* |
| **AnkleLeft** | X_2 | -0.31* |
| **AnkleLeft** | X_3 | -0.27* |
| **AnkleLeft** | X_4 | -0.27* |
| **AnkleLeft** | X_5 | -0.26 |
| **AnkleLeft** | Y_1 | 0.43*** |
| **AnkleLeft** | Y_2 | 0.43*** |
| **AnkleLeft** | Y_3 | 0.27* |
| **AnkleLeft** | Y_4 | 0.27* |
| **AnkleLeft** | Y_5 | 0.26 |
| **AnkleLeft** | Z_1 | 0.42**^c^ |
| **AnkleLeft** | Z_2 | 0.42** |
| **AnkleLeft** | Z_3 | 0.39** |
| **AnkleLeft** | Z_4 | 0.39** |
| **AnkleLeft** | Z_5 | 0.36** |
| **ElbowLeft** | X_1 | -0.25 |
| **ElbowLeft** | X_2 | -0.25 |
| **ElbowLeft** | X_3 | 0.25 |
| **ElbowLeft** | X_4 | -0.20 |
| **ElbowLeft** | X_5 | -0.20 |
| **ElbowLeft** | Y_1 | 0.19 |
| **ElbowLeft** | Y_2 | 0.19 |
| **ElbowLeft** | Y_3 | -0.14 |
| **ElbowLeft** | Y_4 | -0.14 |
| **ElbowLeft** | Y_5 | -0.14 |
| **ElbowLeft** | Z_1 | -0.31* |
| **ElbowLeft** | Z_2 | -0.28* |
| **ElbowLeft** | Z_3 | -0.28* |
| **ElbowLeft** | Z_4 | -0.13 |
| **ElbowLeft** | Z_5 | -0.13 |
| **ElbowRight** | X_1 | 0.15 |
| **ElbowRight** | X_2 | -0.08 |
| **ElbowRight** | X_3 | -0.08 |
| **ElbowRight** | X_4 | -0.07 |
| **ElbowRight** | X_5 | -0.07 |
| **ElbowRight** | Y_1 | 0.32* |
| **ElbowRight** | Y_2 | 0.32* |
| **ElbowRight** | Y_3 | 0.31* |
| **ElbowRight** | Y_4 | 0.31* |
| **ElbowRight** | Y_5 | 0.28* |
| **ElbowRight** | Z_1 | -0.21 |
| **ElbowRight** | Z_2 | -0.21 |
| **ElbowRight** | Z_3 | -0.19 |
| **ElbowRight** | Z_4 | -0.19 |
| **ElbowRight** | Z_5 | -0.18 |
| **FootLeft** | X_1 | -0.30* |
| **FootLeft** | X_2 | -0.30* |
| **FootLeft** | X_3 | -0.26 |
| **FootLeft** | X_4 | -0.26 |
| **FootLeft** | X_5 | -0.25 |
| **FootLeft** | Y_1 | 0.25 |
| **FootLeft** | Y_2 | 0.23 |
| **FootLeft** | Y_3 | 0.23 |
| **FootLeft** | Y_4 | -0.20 |
| **FootLeft** | Y_5 | -0.20 |
| **FootLeft** | Z_1 | 0.33* |
| **FootLeft** | Z_2 | 0.33* |
| **FootLeft** | Z_3 | 0.30* |
| **FootLeft** | Z_4 | 0.30* |
| **FootLeft** | Z_5 | 0.29* |
| **FootRight** | X_1 | -0.24 |
| **FootRight** | X_2 | -0.24 |
| **FootRight** | X_3 | -0.24 |
| **FootRight** | X_4 | -0.24 |
| **FootRight** | X_5 | -0.16 |
| **FootRight** | Y_1 | 0.29* |
| **FootRight** | Y_2 | 0.27* |
| **FootRight** | Y_3 | 0.27* |
| **FootRight** | Y_4 | 0.25 |
| **FootRight** | Y_5 | 0.25 |
| **FootRight** | Z_1 | 0.38** |
| **FootRight** | Z_2 | 0.38** |
| **FootRight** | Z_3 | 0.20 |
| **FootRight** | Z_4 | 0.20 |
| **FootRight** | Z_5 | 0.15 |
| **HandLeft** | X_1 | -0.29* |
| **HandLeft** | X_2 | -0.29* |
| **HandLeft** | X_3 | -0.28* |
| **HandLeft** | X_4 | -0.28* |
| **HandLeft** | X_5 | 0.27* |
| **HandLeft** | Y_1 | 0.44*** |
| **HandLeft** | Y_2 | -0.21 |
| **HandLeft** | Y_3 | -0.21 |
| **HandLeft** | Y_4 | -0.17 |
| **HandLeft** | Y_5 | -0.17 |
| **HandLeft** | Z_1 | 0.18 |
| **HandLeft** | Z_2 | 0.18 |
| **HandLeft** | Z_3 | 0.15 |
| **HandLeft** | Z_4 | 0.15 |
| **HandLeft** | Z_5 | -0.14 |
| **HandRight** | X_1 | -0.11 |
| **HandRight** | X_2 | -0.11 |
| **HandRight** | X_3 | -0.10 |
| **HandRight** | X_4 | -0.10 |
| **HandRight** | X_5 | -0.10 |
| **HandRight** | Y_1 | 0.40** |
| **HandRight** | Y_2 | -0.28* |
| **HandRight** | Y_3 | -0.28* |
| **HandRight** | Y_4 | -0.23 |
| **HandRight** | Y_5 | -0.23 |
| **HandRight** | Z_1 | -0.18 |
| **HandRight** | Z_2 | -0.18 |
| **HandRight** | Z_3 | -0.17 |
| **HandRight** | Z_4 | -0.17 |
| **HandRight** | Z_5 | -0.15 |
| **HandTipLeft** | X_1 | -0.29* |
| **HandTipLeft** | X_2 | -0.29* |
| **HandTipLeft** | X_3 | -0.29* |
| **HandTipLeft** | X_4 | -0.29* |
| **HandTipLeft** | X_5 | 0.25 |
| **HandTipLeft** | Y_1 | 0.38** |
| **HandTipLeft** | Y_2 | 0.23 |
| **HandTipLeft** | Y_3 | 0.23 |
| **HandTipLeft** | Y_4 | 0.18 |
| **HandTipLeft** | Y_5 | 0.18 |
| **HandTipLeft** | Z_1 | 0.18 |
| **HandTipLeft** | Z_2 | 0.18 |
| **HandTipLeft** | Z_3 | 0.17 |
| **HandTipLeft** | Z_4 | 0.17 |
| **HandTipLeft** | Z_5 | 0.17 |
| **HandTipRight** | X_1 | -0.13 |
| **HandTipRight** | X_2 | -0.13 |
| **HandTipRight** | X_3 | -0.12 |
| **HandTipRight** | X_4 | -0.12 |
| **HandTipRight** | X_5 | -0.11 |
| **HandTipRight** | Y_1 | 0.41** |
| **HandTipRight** | Y_2 | 0.22 |
| **HandTipRight** | Y_3 | 0.22 |
| **HandTipRight** | Y_4 | 0.20 |
| **HandTipRight** | Y_5 | 0.20 |
| **HandTipRight** | Z_1 | -0.19 |
| **HandTipRight** | Z_2 | -0.19 |
| **HandTipRight** | Z_3 | -0.16 |
| **HandTipRight** | Z_4 | -0.16 |
| **HandTipRight** | Z_5 | -0.16 |
| **Head** | X_1 | -0.19 |
| **Head** | X_2 | -0.19 |
| **Head** | X_3 | -0.16 |
| **Head** | X_4 | -0.16 |
| **Head** | X_5 | -0.15 |
| **Head** | Y_1 | 0.29* |
| **Head** | Y_2 | 0.29* |
| **Head** | Y_3 | 0.29* |
| **Head** | Y_4 | 0.29* |
| **Head** | Y_5 | 0.27* |
| **Head** | Z_1 | 0.20 |
| **Head** | Z_2 | 0.20 |
| **Head** | Z_3 | 0.19 |
| **Head** | Z_4 | 0.19 |
| **Head** | Z_5 | 0.19 |
| **HipLeft** | X_1 | 0.23 |
| **HipLeft** | X_2 | 0.13 |
| **HipLeft** | X_3 | 0.13 |
| **HipLeft** | X_4 | 0.13 |
| **HipLeft** | X_5 | 0.13 |
| **HipLeft** | Y_1 | -0.24 |
| **HipLeft** | Y_2 | -0.24 |
| **HipLeft** | Y_3 | -0.23 |
| **HipLeft** | Y_4 | -0.23 |
| **HipLeft** | Y_5 | -0.17 |
| **HipLeft** | Z_1 | 0.20 |
| **HipLeft** | Z_2 | 0.20 |
| **HipLeft** | Z_3 | 0.18 |
| **HipLeft** | Z_4 | 0.18 |
| **HipLeft** | Z_5 | 0.17 |
| **HipRight** | X_1 | -0.21 |
| **HipRight** | X_2 | -0.21 |
| **HipRight** | X_3 | -0.15 |
| **HipRight** | X_4 | -0.15 |
| **HipRight** | X_5 | 0.13 |
| **HipRight** | Y_1 | -0.23 |
| **HipRight** | Y_2 | -0.23 |
| **HipRight** | Y_3 | -0.20 |
| **HipRight** | Y_4 | -0.20 |
| **HipRight** | Y_5 | -0.20 |
| **HipRight** | Z_1 | 0.22 |
| **HipRight** | Z_2 | 0.22 |
| **HipRight** | Z_3 | 0.21 |
| **HipRight** | Z_4 | 0.21 |
| **HipRight** | Z_5 | 0.20 |
| **KneeLeft** | X_1 | -0.34** |
| **KneeLeft** | X_2 | -0.34** |
| **KneeLeft** | X_3 | -0.34** |
| **KneeLeft** | X_4 | -0.34** |
| **KneeLeft** | X_5 | -0.19 |
| **KneeLeft** | Y_1 | 0.26 |
| **KneeLeft** | Y_2 | 0.25 |
| **KneeLeft** | Y_3 | 0.25 |
| **KneeLeft** | Y_4 | 0.24 |
| **KneeLeft** | Y_5 | 0.24 |
| **KneeLeft** | Z_1 | 0.40** |
| **KneeLeft** | Z_2 | 0.40** |
| **KneeLeft** | Z_3 | 0.33* |
| **KneeLeft** | Z_4 | 0.33* |
| **KneeLeft** | Z_5 | 0.33* |
| **KneeRight** | X_1 | -0.31* |
| **KneeRight** | X_2 | -0.31* |
| **KneeRight** | X_3 | -0.30* |
| **KneeRight** | X_4 | -0.30* |
| **KneeRight** | X_5 | -0.29* |
| **KneeRight** | Y_1 | -0.35** |
| **KneeRight** | Y_2 | -0.35** |
| **KneeRight** | Y_3 | -0.31* |
| **KneeRight** | Y_4 | -0.31* |
| **KneeRight** | Y_5 | 0.27* |
| **KneeRight** | Z_1 | 0.48*** |
| **KneeRight** | Z_2 | 0.48*** |
| **KneeRight** | Z_3 | 0.36** |
| **KneeRight** | Z_4 | 0.36** |
| **KneeRight** | Z_5 | 0.24 |
| **Neck** | X_1 | -0.21 |
| **Neck** | X_2 | -0.21 |
| **Neck** | X_3 | -0.21 |
| **Neck** | X_4 | -0.21 |
| **Neck** | X_5 | -0.19 |
| **Neck** | Y_1 | 0.21 |
| **Neck** | Y_2 | 0.21 |
| **Neck** | Y_3 | 0.17 |
| **Neck** | Y_4 | 0.17 |
| **Neck** | Y_5 | 0.17 |
| **Neck** | Z_1 | -0.26 |
| **Neck** | Z_2 | -0.26 |
| **Neck** | Z_3 | -0.17 |
| **Neck** | Z_4 | -0.17 |
| **Neck** | Z_5 | 0.14 |
| **ShoulderLeft** | X_1 | 0.23 |
| **ShoulderLeft** | X_2 | -0.22 |
| **ShoulderLeft** | X_3 | -0.22 |
| **ShoulderLeft** | X_4 | -0.22 |
| **ShoulderLeft** | X_5 | -0.22 |
| **ShoulderLeft** | Y_1 | -0.16 |
| **ShoulderLeft** | Y_2 | -0.16 |
| **ShoulderLeft** | Y_3 | 0.14 |
| **ShoulderLeft** | Y_4 | 0.11 |
| **ShoulderLeft** | Y_5 | 0.11 |
| **ShoulderLeft** | Z_1 | -0.24 |
| **ShoulderLeft** | Z_2 | 0.23 |
| **ShoulderLeft** | Z_3 | 0.23 |
| **ShoulderLeft** | Z_4 | 0.23 |
| **ShoulderLeft** | Z_5 | 0.23 |
| **ShoulderRight** | X_1 | 0.15 |
| **ShoulderRight** | X_2 | 0.09 |
| **ShoulderRight** | X_3 | 0.09 |
| **ShoulderRight** | X_4 | 0.08 |
| **ShoulderRight** | X_5 | 0.08 |
| **ShoulderRight** | Y_1 | 0.23 |
| **ShoulderRight** | Y_2 | 0.23 |
| **ShoulderRight** | Y_3 | 0.21 |
| **ShoulderRight** | Y_4 | 0.21 |
| **ShoulderRight** | Y_5 | 0.21 |
| **ShoulderRight** | Z_1 | -0.25 |
| **ShoulderRight** | Z_2 | -0.25 |
| **ShoulderRight** | Z_3 | -0.23 |
| **ShoulderRight** | Z_4 | -0.23 |
| **ShoulderRight** | Z_5 | -0.20 |
| **SpineMid** | X_1 | -0.21 |
| **SpineMid** | X_2 | -0.21 |
| **SpineMid** | X_3 | -0.20 |
| **SpineMid** | X_4 | -0.20 |
| **SpineMid** | X_5 | -0.19 |
| **SpineMid** | Y_1 | 0.20 |
| **SpineMid** | Y_2 | 0.20 |
| **SpineMid** | Y_3 | 0.19 |
| **SpineMid** | Y_4 | 0.19 |
| **SpineMid** | Y_5 | 0.19 |
| **SpineMid** | Z_1 | -0.24 |
| **SpineMid** | Z_2 | -0.24 |
| **SpineMid** | Z_3 | -0.16 |
| **SpineMid** | Z_4 | -0.16 |
| **SpineMid** | Z_5 | 0.11 |
| **SpineShoulder** | X_1 | -0.21 |
| **SpineShoulder** | X_2 | -0.21 |
| **SpineShoulder** | X_3 | -0.21 |
| **SpineShoulder** | X_4 | -0.21 |
| **SpineShoulder** | X_5 | -0.19 |
| **SpineShoulder** | Y_1 | 0.21 |
| **SpineShoulder** | Y_2 | 0.21 |
| **SpineShoulder** | Y_3 | 0.18 |
| **SpineShoulder** | Y_4 | 0.18 |
| **SpineShoulder** | Y_5 | 0.17 |
| **SpineShoulder** | Z_1 | -0.25 |
| **SpineShoulder** | Z_2 | -0.25 |
| **SpineShoulder** | Z_3 | -0.18 |
| **SpineShoulder** | Z_4 | -0.18 |
| **SpineShoulder** | Z_5 | 0.14 |
| **ThumbLeft** | X_1 | -0.35** |
| **ThumbLeft** | X_2 | -0.35** |
| **ThumbLeft** | X_3 | -0.33* |
| **ThumbLeft** | X_4 | -0.33* |
| **ThumbLeft** | X_5 | -0.26 |
| **ThumbLeft** | Y_1 | 0.42** |
| **ThumbLeft** | Y_2 | -0.17 |
| **ThumbLeft** | Y_3 | -0.17 |
| **ThumbLeft** | Y_4 | -0.17 |
| **ThumbLeft** | Y_5 | -0.17 |
| **ThumbLeft** | Z_1 | 0.25 |
| **ThumbLeft** | Z_2 | 0.25 |
| **ThumbLeft** | Z_3 | 0.18 |
| **ThumbLeft** | Z_4 | 0.18 |
| **ThumbLeft** | Z_5 | 0.16 |
| **ThumbRight** | X_1 | -0.12 |
| **ThumbRight** | X_2 | -0.12 |
| **ThumbRight** | X_3 | -0.11 |
| **ThumbRight** | X_4 | -0.11 |
| **ThumbRight** | X_5 | -0.10 |
| **ThumbRight** | Y_1 | 0.41** |
| **ThumbRight** | Y_2 | -0.23 |
| **ThumbRight** | Y_3 | -0.23 |
| **ThumbRight** | Y_4 | 0.22 |
| **ThumbRight** | Y_5 | 0.22 |
| **ThumbRight** | Z_1 | -0.21 |
| **ThumbRight** | Z_2 | -0.21 |
| **ThumbRight** | Z_3 | -0.15 |
| **ThumbRight** | Z_4 | -0.15 |
| **ThumbRight** | Z_5 | -0.12 |
| **WristLeft** | X_1 | 0.27* |
| **WristLeft** | X_2 | -0.27* |
| **WristLeft** | X_3 | -0.27* |
| **WristLeft** | X_4 | -0.26 |
| **WristLeft** | X_5 | -0.26 |
| **WristLeft** | Y_1 | -0.15 |
| **WristLeft** | Y_2 | -0.15 |
| **WristLeft** | Y_3 | -0.14 |
| **WristLeft** | Y_4 | -0.13 |
| **WristLeft** | Y_5 | -0.13 |
| **WristLeft** | Z_1 | -0.16 |
| **WristLeft** | Z_2 | -0.16 |
| **WristLeft** | Z_3 | -0.16 |
| **WristLeft** | Z_4 | -0.13 |
| **WristLeft** | Z_5 | -0.13 |
| **WristRight** | X_1 | -0.10 |
| **WristRight** | X_2 | -0.10 |
| **WristRight** | X_3 | -0.10 |
| **WristRight** | X_4 | -0.10 |
| **WristRight** | X_5 | -0.08 |
| **WristRight** | Y_1 | 0.24 |
| **WristRight** | Y_2 | 0.24 |
| **WristRight** | Y_3 | -0.19 |
| **WristRight** | Y_4 | 0.19 |
| **WristRight** | Y_5 | 0.19 |
| **WristRight** | Z_1 | -0.18 |
| **WristRight** | Z_2 | -0.18 |
| **WristRight** | Z_3 | -0.18 |
| **WristRight** | Z_4 | -0.18 |
| **WristRight** | Z_5 | -0.17 |

^a^ * p < 0.05

^b^ *** p < 0.001

^c^ ** p < 0.01
