# Supplementary material for: Gait can reveal sleep quality with machine learning models
Source: PLoS One. 2019 Sep 25;14(9):e0223012. doi: 10.1371/journal.pone.0223012 (PMC6760789; doi:10.1371/journal.pone.0223012)
Supplement: S1 File — (DOCX) [file pone.0223012.s002.docx]

GaussianProcesses：

batchSize: 100; numDecimalPlaces: 2; noise: 1.0; kernel: PolyKernel.

LinearRegression:

batchSize: 100; ridge: 1.0E-8; arreibuteSelectionMethod: M5 method; numDecimalPlaces: 4.

SimpleLinearRegression:

batchSize: 100; numDecimalPlaces: 4.

E-SVR:

batchSize: 100; kernelType: polynomial; loss: 0.1; cost: 1; degree: 3.

N-SVR:

batchSize: 100; kernelType: radial basis function; loss: 0.1; cost: 1; degree: 3.
